# Lack of canonical activities of connexins in highly aggressive human prostate cancer cells

**DOI:** 10.1186/s40659-024-00565-3

**Published:** 2024-12-19

**Authors:** Catalina Asencio, Loreto Véliz, Emilia Flores-Faúndez, Lorena Azócar, Carolina E. Echeverría, Verónica Torres-Estay, Viviana Orellana, Catalina Ramírez-Santelices, Paula Sotomayor, Jorge Cancino, Bredford Kerr, Ainoa Fernandez-Olivares, Mauricio A. Retamal, Juan C. Sáez, Alejandro S. Godoy

**Affiliations:** 1 Facultad de Ciencias Biológicas, Pontificia Universidad Católica de Chile, Santiago, Chile; 2 Department of Urology, Pontificia Universidad Católica de Chile, Santiago, Chile; 3 Centro de Biología Celular y Biomedicina CEBICEM, Facultad de Medicina y Ciencia, Universidad San Sebastián, Lota 2465, Providencia., Santiago, 7510156 Chile; 4 Escuela de Química y Farmacia, Facultad de Medicina y Ciencia, Universidad San Sebastián, Santiago, Chile; 5 Programa de Comunicación Celular en Cáncer, Instituto de Ciencias e Innovación en Medicina, Facultad de Medicina, Clínica Alemana Universidad del Desarrollo, Santiago, 7610634, Chile; 6 Centro Interdisciplinario de Neurociencias de Valparaíso, Instituto de Neurociencia, Universidad de Valparaíso, Valparaíso, Chile; 7 Department of Medicine, NYU Grossman School of Medicine, New York, NY USA; 8 Department of Urology, Roswell Park Comprehensive Cancer Center, Buffalo, NY USA

**Keywords:** Prostate cancer, Connexins, Gap junctions, Hemichannels

## Abstract

Connexins (Cxs) have the ability to form channels that allow the exchange of ions/metabolites between adjacent cells (gap junction channels, GJC) or between the intra- and extra-cellular compartments (hemichannels, HC). Cxs were initially classified as tumor suppressors. However, more recently, it has been shown that Cxs exert anti- and pro-tumorigenic effects depending on the cell and tissue context. In prostate cancer (PCa), the expression and functionality of Cxs remain highly controversial. Here, we analyzed the expression pattern of Cx26, Cx32, Cx37, Cx40, Cx43 and Cx45 in PCa cell lines with increasing levels of tumor aggressiveness (LNCaP < LNCaP-C4-2 < Du-145 < PC-3). In addition, GJ and HC activities were evaluated in the PCa cell lines using dye coupling and dye uptake assays, respectively. Lastly, the cellular localization of Cx26, Cx32, and Cx43 was analyzed in LNCaP and PC-3 cell lines using immunofluorescence analyses. Our results showed a positive association between the mRNA levels of Cx26, Cx37 and Cx45 and the degree of aggressiveness of PCa cells, a negative association in the case of Cx32 and Cx43, and no clear pattern for Cx40. At the protein level, a negative relationship between the expression of Cx26, Cx32 and Cx43 and the degree of aggressiveness of PCa cell lines was observed. No significant differences were observed for the expression of Cx37, Cx40, and Cx45 in PCa cell lines. At the functional level, only LNCaP cells showed moderate GJ activity and LNCaP and LNCaP-C4-2 cells showed HC activity. Immunofluorescence analyses confirmed that the majority of Cx26, Cx32, and Cx43 expression was localized in the cytoplasm of both LNCaP and PC3 cell lines. This data indicated that GJ and HC activities were moderately detected only in the less aggressive PCa cells, which suggest that Cxs expression in highly aggressive PCa cells could be associated to channel-independent roles.

## Introduction

Connexins (Cxs) represent a highly conserved family of proteins, of at least 21 members, which are differentially expressed in almost all types of vertebrate cells [1, 2]. These proteins are named according to their predicted molecular weight, from the smallest (Cx23, 23 kDa) to the largest (Cx62, 62 kDa) connexin [3]. At the molecular level, six Cxs oligomerize to form a transmembrane structure called hemichannel (HC) or connexon [4]. These structures allow passive flow of ions, metabolites, such as glucose, and signaling molecules, like ATP and PGE_2_, between the intracellular and the extracellular compartments [3, 5, 6]. Two HCs from adjacent cells can join through their extracellular loops to form an intercellular gap junction channel (GJC) and a cluster of a variable number of the latter form the membrane specialization called gap junction plaques [4]. Through the GJC, cells can rapidly, and most frequently bi-directionally, exchange a variety of cytosolic molecules by simple diffusion in a process known as gap junction mediated intercellular communication (GJIC) [1]. This type of communication is essential for various cell functions, such as cell growth, proliferation and differentiation, among many others [7, 8].

In 1966, Loewenstein and Kanno [9] demonstrated that the GJ-dependent electrical coupling between healthy hepatocytes was lost in liver tumor cells. This result was initially interpreted as a direct link between lack of cell-cell coupling and lack of control of cell growth. Subsequent studies in human thyroid and mammalian malignancies supported the hypothesis that the loss of GJIC was a characteristic of cancer cells [10, 11]. This view has been changing over the last 10 years. For instance, Cxs have been involved in facilitating tumor progression in the later stages of the disease by actively promoting tumor cell migration and invasion, extravasation and metastasis [12–15].

Among all human malignancies, prostate cancer (PCa) represents the second leading cause of cancer-related death in American and European men [16–18]. Interestingly, previous evidence has shown that the dysfunction of GJIC is implicated in the development and progression of PCa [19–21]. Tsai et al. [22] showed, in a limited set of paired benign and malignant human prostate tissues, a reduction in Cx43 levels in neoplastic tissues compared to their benign counterparts. In addition, Habermann et al. [23]. showed that Cx32 expression was significantly decreased in PCa compared to benign tissues, with a remarkable decrease in poorly differentiated PCa specimens. Analysis of the distribution patterns of Cx32 and Cx43 in human prostate tumors with different histological grades showed that, in well-differentiated prostate tumors, these Cxs were located in areas of cell-cell contact. However, as prostate tumors progressed to more undifferentiated states, Cx32 and Cx43 were detected in the cytoplasmic compartment, followed by eventual loss of the proteins in advanced stages [24]. Also, Benko et al. [25] and Xu et al. [26] reported that a significant reduction or loss of Cx43 expression showed a negative correlation with poor prognosis characteristics, such as progression time, preoperative PSA, and biochemical recurrence free-survival (BFS) after radical prostatectomy.

Despite these evidences in human clinical specimens, the in vitro studies are less conclusive and, in several cases, highly controversial. Mehta et al. [27] showed that ALVA-31, ALVA-41, ALVA-55 and PC-3 cell lines only expressed Cx43, while human prostate epithelial cells established from benign donors expressed Cx32 and Cx40. On the contrary, other studies [15, 28] showed that the PCa cell lines, LNCaP and PC-3, expressed Cx26 and Cx45 besides Cx43. Studies on HC/GJIC activity and their biological impact on in vitro models of PCa are rarer and even less conclusive. An in vitro study using the benign prostate epithelial cell lines, NPE-1 and 2, and the PCa cell lines PC-3, DU-145, LNCaP, and ALVA indicated that GJIC was reduced or not detected in malignant when compared to benign prostate epithelial cells [27]. At the biological level, an increase in the expression of Cx43 was correlated with a reduction in adhesion and invasion capacities in PC-3 cells. On the contrary, Cx43 increased cellular aggressiveness in LNCaP cells [29]. Two independent studies in PCa cell lines, LNCaP and PC-3, reported that the expression of Cx26 and Cx43 were positively correlated with an increase of their malignancy [15, 28]. Furthermore, the treatment with GJ inhibitors significantly reduced cell migration and invasion capacities in these cell lines. These observations were also supported by Tate et al. [28], where they showed a direct association between Cx26 and focal adhesion kinase (FAK) in LNCaP and PC-3 cells and Zhang et al. [15], who reported that suppression of Cx43 expression in PC-3 cells, using a shRNA assay, inhibited PC-3 cell migration and invasion.

For connexons, a mechanism involving Cx43 HC-mediated autocrine regulation in breast cancer cells has been described recently, which indicated that release of nucleosides through HCs is essential for the breast cancer cell invasion [30]. Zhou et al. [31], demonstrated an inhibitory role of Cx43 HCs, present in osteocytes, over anchorage-independent growth, migration and invasion of MDA-MB-231 human breast cancer cells. However, so far, no information related to HC activity or functional state has been reported in PCa cells.

In summary, it is increasingly evident that Cxs can differentially affect PCa cell biology by selectively promoting PCa progression in vivo and in vitro. However, three major points within this topic remain unanswered, or at least, highly controversial, which we intend to address in this study: (1) A clear identification of the expression pattern of Cxs, and their relationship with the degree of aggressiveness, in PCa cell lines, (2) The determination whether there is indeed a GJIC deficiency in PCa cell lines or if it is related to the degree of aggressiveness of malignant prostate cells, and (3) The study of a possible relationship between HC activity and the degree of aggressiveness of PCa cell lines.

## Materials and methods

### Cell Culture

Human PCa cell lines LNCaP, LNCaP-C4-2, Du-145 and PC-3 were obtained from the American Type Culture Collection (Rockvile, MD, USA). PCa cell lines were grown in standard Roswell Park Memorial Institute-1640 (RPMI-1640) media, supplemented with 10% heat-inactivated fetal bovine serum (FBS), 1% antibiotic penicillin and streptomycin and 1nM dihydrotestosterone (DHT). All the cells were maintained under a humidified incubator (37.0 °C, 5% CO_2_) [32].

### RNA extraction and real-time reverse transcription polymerase chain reaction

Total RNA was extracted with RNAzol^®^ according to manufacturer’s protocols from PCa cell lines. Aliquots of 1.0 µg of total RNA were reversed-transcribed into cDNA according to the instructions of the manufactures (Promega). Real time polymerase chain reaction (RT-PCR) was performed using Fast Sybr Green Mix (CO110820, Applied Biosystems). The relative amount of specific mRNA was normalized to 18S. All RT-PCRs were run in triplicate and were performed over 40 cycles. Analysis of the results was carried out by using the 2^−ΔCT^ method [33]. All extracted RNA samples from each cell line equal or close to 2 ratio (260/280nm) were used. The primers used were as follows: 18S, 5’-GCCGCTAGAGGTGAAATTCTTGGA-3’ (forward) and 5’-ATCGCCAGTCGGCATCGTTTAT-3’ (reverse), Cx26, 5’-CGTGTGCTACGATCACTACTT-3’ (forward) and 5’-CTTCCTCTTCTTCTCATGTCTCC-3’ (reverse), Cx32 5’-GCTCACCAGCAACACATAGA-3’ (forward) and 5’-TCCCTGAGATGTGGACCTT-3’ (reverse), Cx43 5’-AGCAGTCTGCCTTTCGTTGTA-3’ (forward) and 5’-GATTGGGAAAGACTTGTCATAGCAG-3’ (reverse), Cx37 5’-GCAGGTTGTCCTCAAACCCT-3’ (forward) and 5’-GTCTGTGCGATCAAGGCTGA-3’ (reverse), Cx40 5’-CGAGCAAGTACGAGGTCAGG-3’ (forward) and 5’-GAGACTCCATTGGGCACCTC-3’ (reverse), Cx45 5’-GAAGAGTGCCCCTGGTGTTT-3’ (forward) and 5’-AGCTGACGCGTTATCCAGAC-3’ (reverse).

### Western blot

Cells were rinsed three times with chilled phosphate-buffered saline (PBS). Subsequently, RIPA lysis buffer [50 mM Tris-HCl (pH 8), 150 mM NaCl, 1% Triton X-100, Sodium Deoxycholate, and 0.1% SDS] supplemented with protease and phosphatase inhibitors (Roche) was added to the cell culture, followed by scraping using a rubber policeman. The cell suspension was immediately sonicated on ice. The protein concentration in the resulting cell homogenate was determined using a Qubit protein assay kit (ThermoFisher Scientific) and measured with a Qubit 3.0 Fluorometer (ThermoFisher Scientific). Then, 4x sample buffer (Invitrogen) was added to the cell homogenate, and the mixture was heated at 95 °C for 3 min. After that, samples were either stored at -80 °C or loaded (40–50 µg of protein) onto a 10% NuPAGE gel (ThermoFisher Scientific) and electro-transferred to a PVDF membrane using an iBlot gel transfer device (ThermoFisher Scientific). Following transfer, the membranes were blocked in 5% bovine serum albumin (BSA) in Tris-buffered saline containing 0.1% Tween 20 (TBS-T) for 1 h at room temperature. They were then probed overnight at 4 °C with primary antibodies against anti-Cx26 (1:1,000, Thermo Scientific), anti-Cx32 (1:500, Invitrogen, USA), anti-Cx43 (1:800, Thermo Scientific), anti-Cx37 (1:1,000, Alpha Diagnostics Int.), anti-Cx40 (1:500, Invitrogen), anti-Cx45 (1:1,000, Santa Cruz Biotechnology), and anti-mouse β-actin (1:10,000, Sigma Aldrich). Next day, the membranes were washed three times with TBS-T and then incubated with horseradish-peroxidase-conjugated secondary antibody for 1 h at room temperature. After three additional washes with TBS-T, the membranes were subjected to chemiluminescence detection using Super SignalTM West Pico Plus (ThermoFisher Scientific, Massachusetts, USA) as per the manufacturer’s protocol. The chemiluminescent signals were visualized using LI-COR C-Digit chemiluminescence Western Blot Scanner systems (LI-COR, Inc, Lincoln, USA) [33]. 

### Dye coupling tests

PCa cells were cultured on glass coverslips and placed on recording medium (HCO_3_-free F-12 medium buffered with 10 mM HEPES, pH 7.2). Cell-to-cell transfer mediated by JGs was tested by evaluating the Lucifer yellow (LY) (-2 charge, 443 kDa) and Dapi (+ 1 charge, 279 kDa) transfer to neighboring cells. Singles cells were microinjected with a glass micropipette filled with 5 µM of LY or DAPI in a recording medium having 200 µM La^+ 3^ to prevent leakage of the microinjected dye through HCs. Fluorescent cells were observed using a Nikon Diaphot inverted microscope equipped with epifluorescence illumination (xenon arc lamp) and Nikon BV-IA filter for LY and DAPI (green emission). Photomicrographs were obtained using a CCD monochromatic camera (CFW-1310 M; Scion; Frederick, MD, USA). Three or five minutes after injection of the LY or DAPI dye, respectively, the cells were observed to determine whether transfer of the dye occurred. The coupling index was calculated as the average number of cells to which the dye was transferred, divided by the number of positive cases. The incidence of coupling was rated as the percentage of injections that resulted in the transfer of the dye from the injected cell to more than one neighboring cell. Three experiments were performed for each cell line and dye coupling was tested by injecting a minimum of 10 cells per experiment.

### Dye Uptake

The activity of HCs was evaluated using the dye uptake method [34]. PCa cells were placed on glass slides and washed twice with recording solution (in mM: NaCl (148); KCl (5); CaCl_2_ (1.8); MgCl_2_ (1); glucose (5); HEPES (5), pH = 7.4). Cells were placed in an inverted microscope (Nikon Eclipse Ti) equipped with LED-based fluorescence and basal dye uptake was measured in cells bathed in a recording solution containing 10 µM DAPI. In order to maximize HC opening, cells were placed in recoding media containing DAPI but without divalent cations (divalent cation-free solution; DCFS) plus 5 mM EGTA. To confirm that DAPI uptake was mediated by HCs they were blocked by using lanthanum ion (La^+ 3^, 200 µM) added at time 0. Time-lapse experiments were recorded every 20 s for 10 min. Then, fluorescence intensity was measured in at least 16 different cells aleatory chosen, using the NIS-Elements software. Dye uptake rates were calculated with Microsoft Excel software and expressed as AU/min. The microscope and camera setting were kept constant in all experiments. The fluorescence of DAPI intercalated to nucleic acids was recorded in regions of interest that corresponded to nuclei of cells by using a Nikon Eclipse Ti inverted microscope (Japan) with NIS-Elements software acquisition, while image processing was performed with ImageJ 1.46r software (National Institute of Health).

### Immunofluorescence

To determine the cellular localization of Cx26, Cx32, and Cx43 in PCa cells, we used immunofluorescence staining combined with confocal laser scanning microscopy (Leica SP8) following established protocols [35]. Briefly, LNCaP and PC-3 cells were seeded on poly-L-lysine-coated slides, stained with biotinylated concanavalin A (1:400, Vector Labs) for 30 minutes at 37°C, and the staining was detected using 568 Alexa Fluor-conjugated streptavidin (1:400, Thermo Fisher Scientific). The cells were then fixed in 4% paraformaldehyde for 30 minutes and permeabilized with 0.2% Triton X-100 for 10 minutes. After fixation and permeabilization, cells were blocked with 50 mM NH_4_Cl for 5 minutes (to neutralize free aldehyde groups) and 3% w/v BSA for 30 minutes (to prevent nonspecific antibody binding). Following blocking, the cells were incubated overnight at 4°C with anti-Cx26 (1:100, Invitrogen), anti-Cx32 (1:100, Invitrogen), or anti-Cx43 (1:100, Invitrogen) antibodies. The immunostaining was visualized by incubating the cells with Alexa Fluor 488-conjugated goat anti-rabbit IgG (1:100, Invitrogen) for 30 minutes at 37°C. Nuclei were counterstained with 4’,6-diamidino-2-phenylindole dihydrochloride (DAPI) (1:50000, Sigma-Aldrich). Lastly, the cells were photographed, and 10 independent images per condition were analyzed using the ImageJ software.

### Statistical analysis

All data were presented as the mean ± standard error mean (SEM). Statistical analysis was performed by using one-way analysis of variance (ANOVA) followed by Tukey’s multiple comparison test and Mann Whitney test. *P < 0.05, **P < 0.001, and ***P < 0.0001 were considered to be significant.

## Results

### Connexins 26, 32, 37 40, 43 and 45 mRNA expression in prostate cancer cell lines

**Fig. 1:**
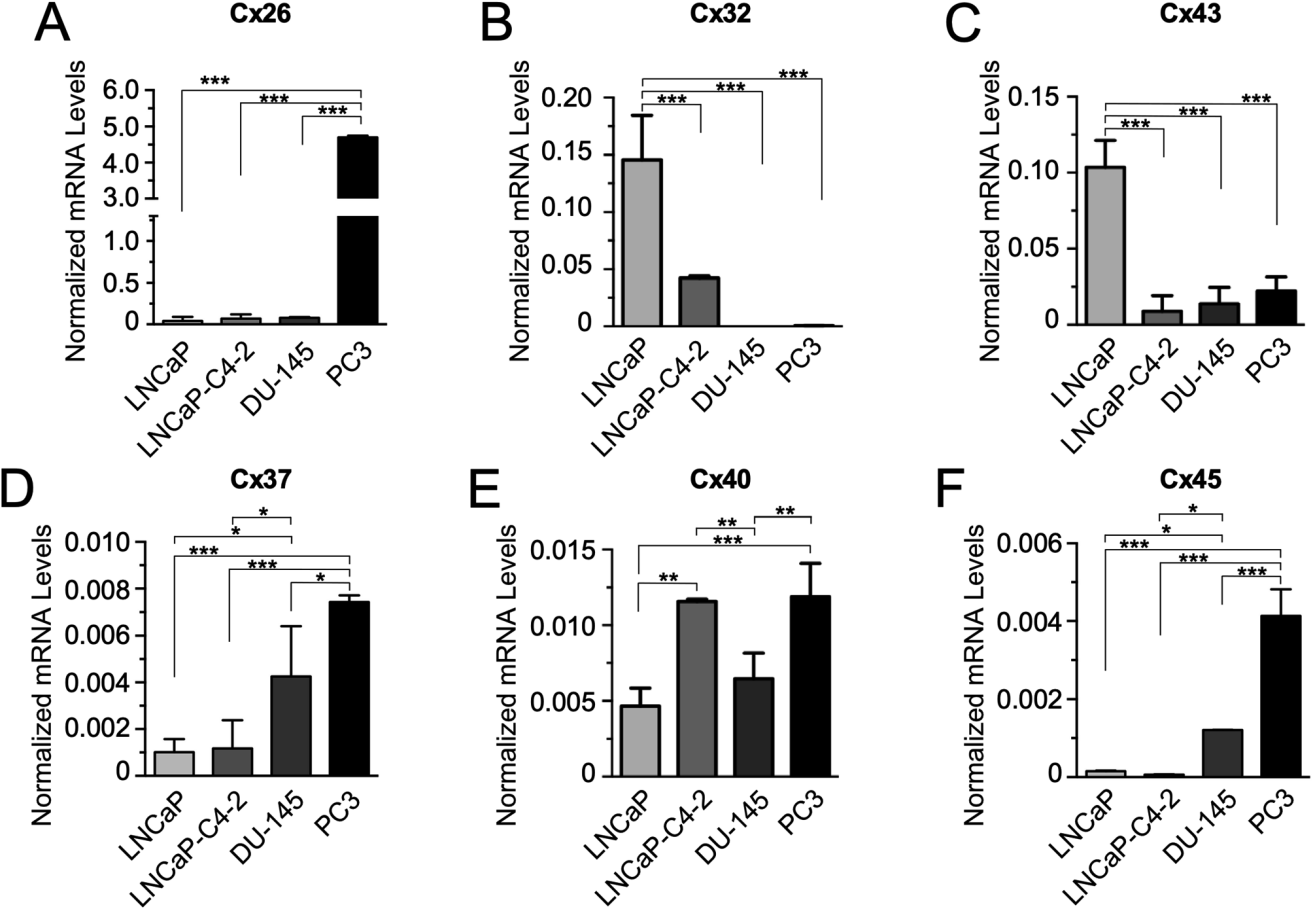
Amount of mRNA of different connexin isoforms in human prostate cancer cell lines. The amount of mRNA of Cx26 (**A**), Cx32 (**B**), Cx43 (**C**), Cx37 (**D**), Cx40 (**E**) and Cx 45 (**F**) was determined in LNCaP, LNCaP-C4-2, DU-145 and PC-3 PCa cell lines by qRT-PCR. 18 S rRNA expression was used to normalize data (2^− deltaCT^). One-way ANOVA followed by Tukey’s tests were utilized for statistical analyses. * = *P* < 0.05, ** = *P* < 0.001, *** = *P* < 0.0001. Each bar represents the mean ± SD of 3 independent experiments, each one performed in triplicate

In order to determine the level of expression of different Cx isoforms and to assess whether their expression have any relationship with the degree of aggressiveness of PCa cell lines, qRT-PCR analyses were performed to measure the relative amount of Cx26, Cx32, Cx37, Cx40, Cx43 and Cx45 mRNAs in four cell lines with increasing degree of tumor aggressiveness, as follows: LNCaP < LNCaP-C4-2 < Du-145 < PC-3. Our results indicate that Cx26, Cx37 and Cx45 showed the highest amount of mRNA in the highly aggressive PC-3 cell line (Fig. 1A, D, and F). On the contrary, Cx32 and Cx43 showed the highest amount of mRNA in the low aggressive LNCaP PCa cell line (Fig. 1B-C), whereas, the relative amount of Cx40 mRNA showed no clear relationship with the degree of aggressiveness of PCa cells. There were only significant differences in the amount of Cx40 mRNA when comparing PC-3 and LNCaP-C4-2 with LNCaP cells (Fig. 1E). Together, our results indicated that there was a positive relationship between the amount of Cx26, Cx37 and Cx45 mRNAs, and a negative relationship between the amount of Cx32 and Cx43 mRNAs, with the degree of aggressiveness of PCa cell lines. In addition, these results suggest that some Cx isoforms, such as Cx26, Cx37 and Cx45, could have a potential inductive role in processes related to cellular aggressiveness in PCa cells.

### Connexins 26, 32, 37 40, 43 and 45 protein expression in prostate cancer cell lines

**Fig. 2:**
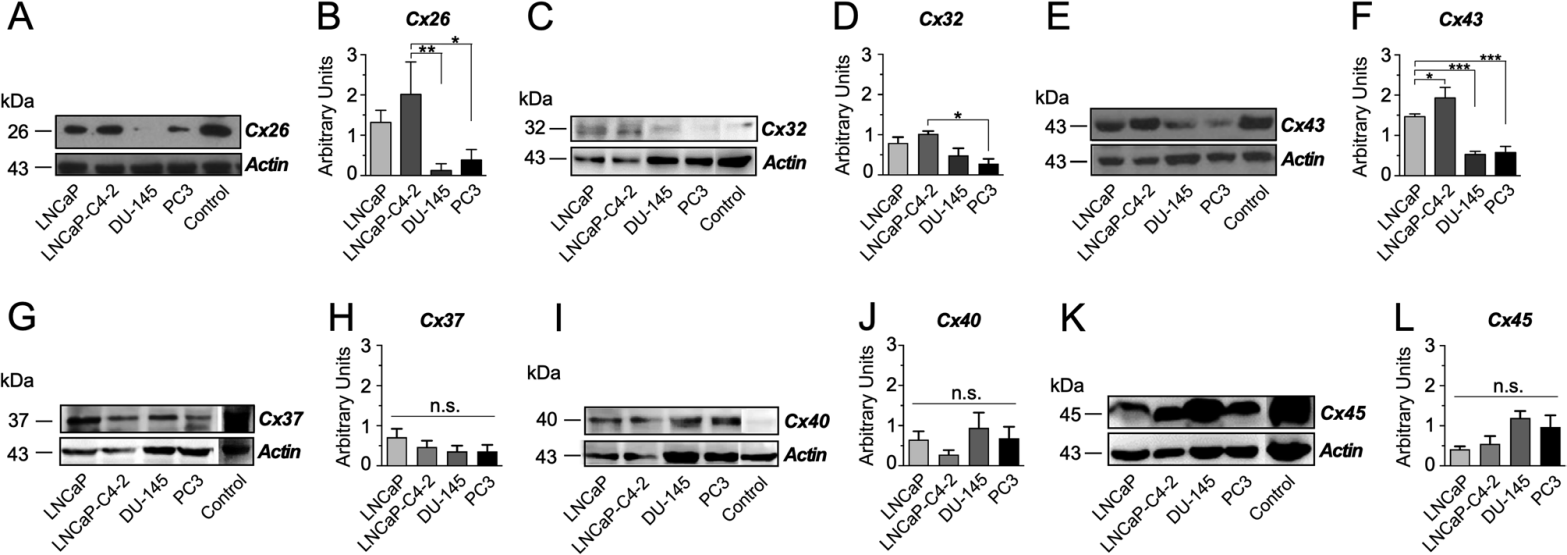
Relative amount of connexin proteins in human prostate cancer cell lines. The relative amount of Cx26 (**A**), Cx32 (**C**), Cx43 (**E**), Cx37 (**G**), Cx40 (**I**) and Cx45 (**K**) protein was analyzed in LNCaP, LNCaP-C4-2, DU-145 and PC-3 PCa cell lines using western blot analysis. β-actin (Actin) was used as a loading control. MDA-MB-231 cell line protein extract was utilized for positive controls. The densitometric analyses of the western blots for Cx26 (**B**), Cx32 (**D**) and Cx43 (**F**) Cx37 (**H**), Cx40 (**J**) and Cx45 (**L**) were carried out using ImageJ software. One-way ANOVA followed by Tukey’s tests were utilized for statistical analyses. * = *P* < 0.05, ** = *P* < 0.001. Each bar represents the mean ± SD of 3 independent experiments

In order to confirm our previous results at the protein level, we studied the relative amount of Cx26, Cx32, Cx37, Cx40, Cx43 and Cx45 protein in PCa cell lines. Contrary to what was observed using qRT-PCR, in the less aggressive PCa cell lines, LNCaP and LNCaP-C4-2, Cx26 showed a higher amount of protein when compared to Du-145 and PC-3 cell lines (Fig. 2A-B). On the other hand, in accordance with the qRT-PCR analysis, Cx32 and Cx43 showed a negative association with the degree of aggressiveness in PCa cell lines (Fig. 2C-F). Also, protein expression of Cx37 (Fig. 2G-H) and Cx45 (Fig. 2K-L) showed a tendency to be negatively and positively associated with the degree of aggressiveness in PCa cell lines, respectively, but no statistical significance was achieved. Lastly, protein expression of Cx40 showed no clear association with the degree of aggressiveness in PCa cell lines (Fig. 2I-J). In general, our results indicated, firstly, that there is no direct correspondence between mRNA levels and protein expression for some of the connexins analyzed, a fact that has also been reported for Cxs 26, 32, 37, 43 and 45 in myeloid leukemia cells [36] and Cx40 in HUVEC cells exposed to shear stress [37]. Secondly, our results demonstrated that, at least at the protein level, Cx26, Cx32 and Cx43, were negatively associated with degree of aggressiveness in PCa cell lines. The protein expression levels of all other connexins (Cx37, Cx40, and Cx45) analyzed showed no a significant association with the degree of aggressiveness of PCa cell lines.

### Gap junctions functionality in prostate Cancer cell lines

**Fig. 3:**
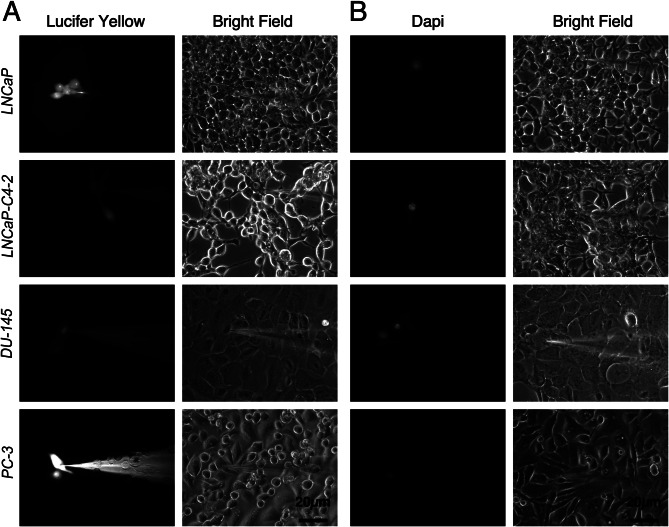
Dye coupling analyses in human prostate cancer cell lines. Lucifer yellow (**A**) or DAPI (**B**) were microinjected in LNCaP, LNCaP-C4-2, Du-145 and PC-3 PCa cells. Left panels show the fluorescence of Lucifer yellow and DAPI and the right panels represent the corresponding phase contrast images. Black bars: 20 μm

**Fig. 4:**
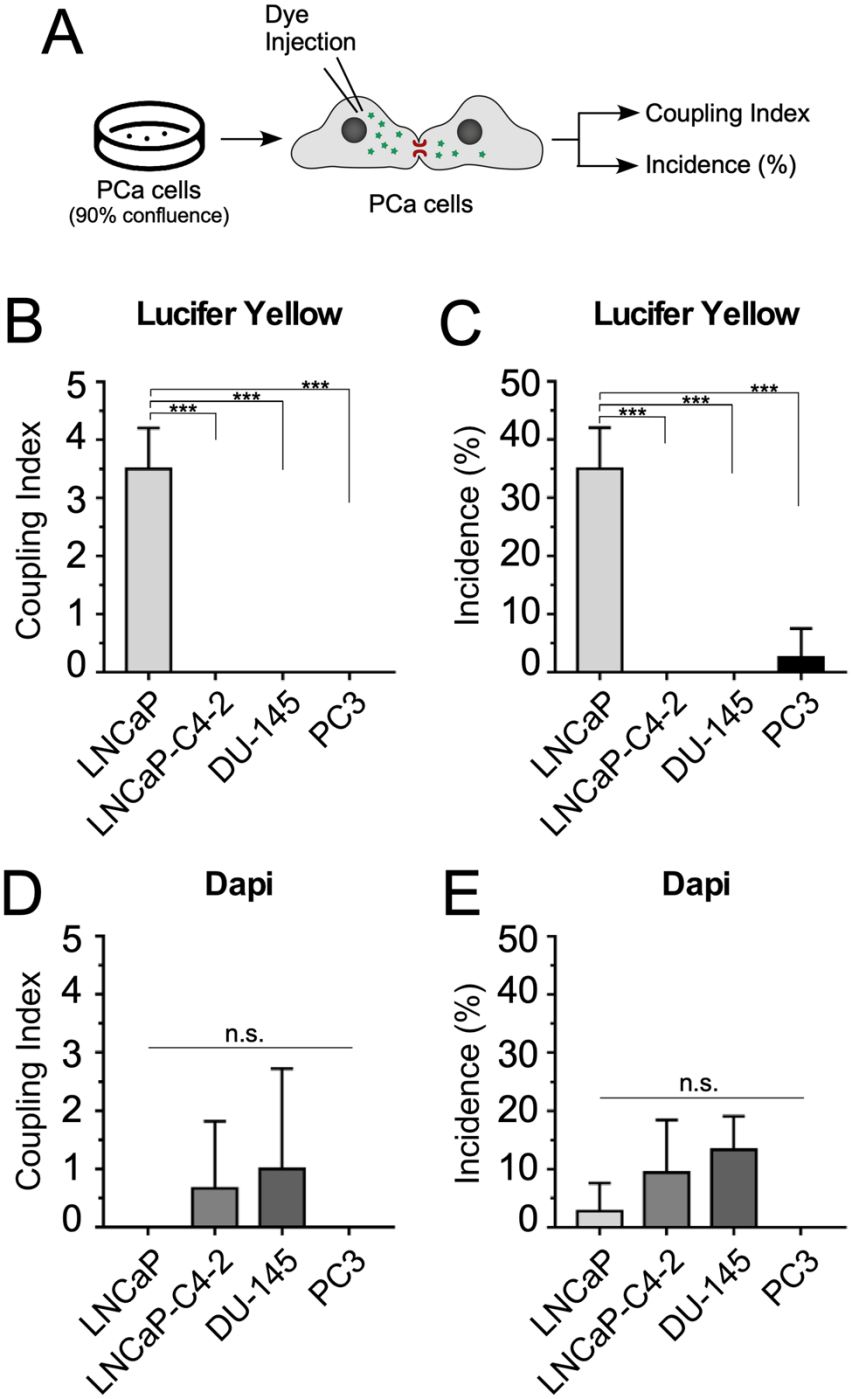
Gap junction activity in human prostate cancer cell lines. (**A**) Schematic representation of the GJC activity measurements. The coupling index (**B** and **D**) and the incidence of coupling (**C** and **E**) were evaluated in confluent cultures of LNCaP, LNCaP-C4-2, Du-145 and PC-3 PCa cell lines using the LY or DAPI. Each bar represents the mean ± SD of 3 independent experiments. For each experiment, the dye (Lucifer yellow or DAPI) was microinjected in at least 10 different cells. One-way ANOVA followed by Tukey’s tests were utilized for statistical analyses. *** = *P* < 0.001. n.s. = not statistically significant

To analyze whether the PCa cell lines have functional GJC and whether their activity is related to the degree of aggressiveness of PCa cell lines, the GJIC was evaluated using the dye coupling method. Figure 3 shows representative images of intercellular transfer of the LY (Fig. 3A) and DAPI (Fig. 3B) in LNCaP, LNCaP-C4-2, Du-145 and PC-3 PCa cell lines. From all PCa cell lines analyzed, absence to low levels of dye transfer was observed in LNCaP-C4-2, Du-145 and PC-3 PCa cell lines. Interestingly, only the less aggressive LNCaP cell line showed moderate LY dye transfer. Specifically, LNCaP cells showed a coupling index (number of cells coupled) of 3.5 for LY (Fig. 4A) and an incidence of dye coupling (percentage of cells coupled to more than one cell) of 35% (Fig. 4B), being significantly higher than the other PCa cell lines analyzed. With DAPI, no dye transfer was observed in the majority of PCa cell lines analyzed (Fig. 3B) and only a weak dye transfer was observed in LNCaP-C4-2 and DU-145 cells (Fig. 4D-E). Interestingly, LNCaP cells exhibited a difference in transfer capacity that depended on the type of dye used (LY vs. DAPI), which was reflected as a higher coupling index and percentage of incidence when using LY when compared to DAPI (Fig. 4).

### Hemichannel activity in prostate Cancer cell lines

**Fig. 5:**
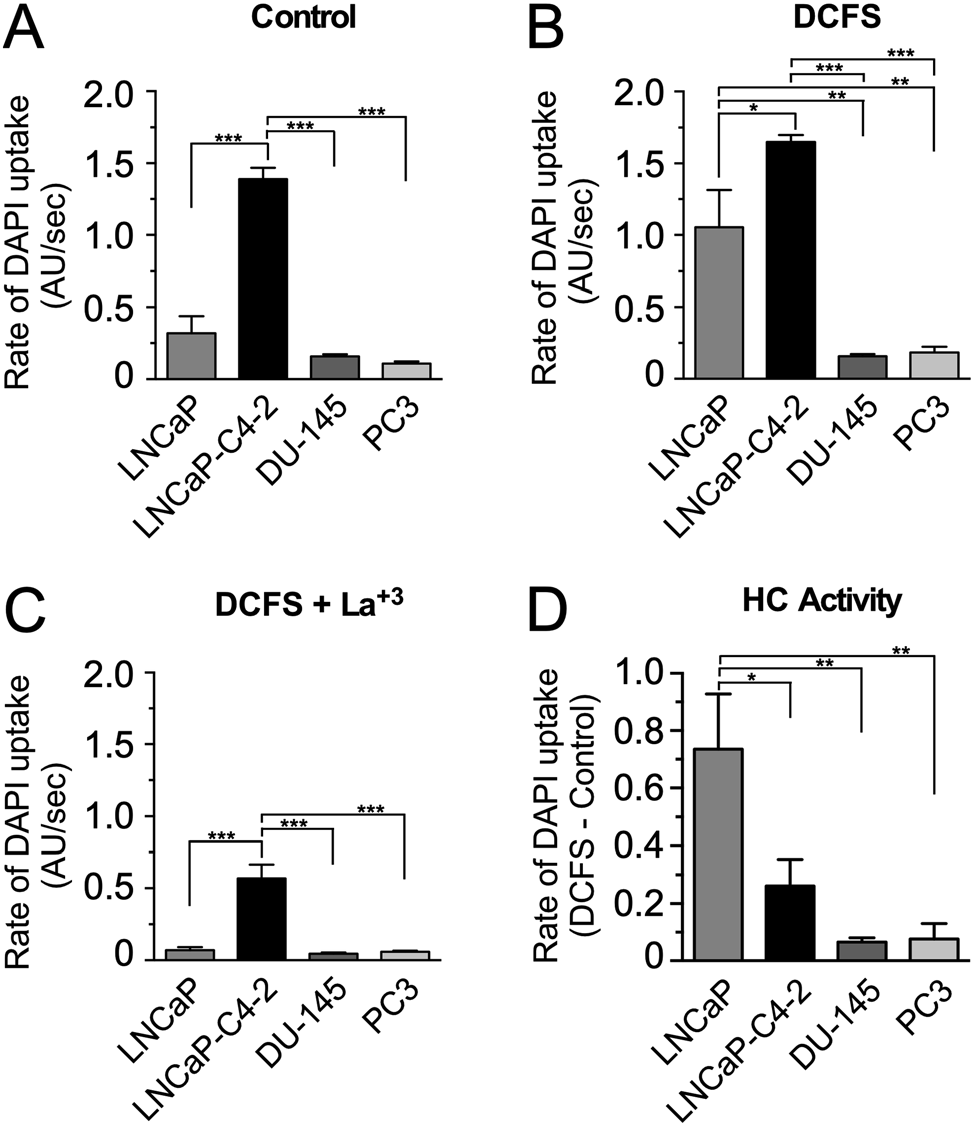
Dye uptake in human prostate cancer cell lines. DAPI (5 µM) uptake was measured every 15 s in LNCaP, LNCaP C4-2, Du-145 and PC-3 cell line cultures. Fluorescence intensity due to DAPI uptake was expressed as arbitrary units (AU). After 5 min, PCa cells were exposed to a medium without divalent cations to stimulate the opening of hemichannels. At the end of the experiment, La^+ 3^ (200 µM) was added to the recording solution to block the hemichannels. (**A**-**D**) DAPI uptake rate in PCa cell lines under basal conditions, under hemichannel stimulation and blockade. (**D**) Relative DAPI uptake (uptake rate under stimulus / basal uptake rate) in PCa cell lines. In each experiment the fluorescence intensity was measured over time in at least 20 cells. Each bar represents the average ± SD of 3 independent experiments, each one performed in triplicate. One-way ANOVA followed by Tukey’s tests were utilized for statistical analyses. * = *P* < 0.05, ** = *P* < 0.01, *** = *P* < 0.001

Lastly, we analyzed whether PCa cell lines possess functional hemichannels using the dye (Dapi) uptake technique (Fig. 5). First, DAPI uptake was measured as fluorescence intensity of the cells estimated under basal (Control, recording medium only) conditions. Under these conditions, almost no dye uptake was observed in all PCa cells lines analyzed, with the exception of LNCaP-C4-2 cells, which presented a high level of basal DAPI uptake rate (Fig. 5A). Then, the hemichannel opening was promoted by incubating the PCa cell lines on a divalent cations-free solution (DCFS) (Fig. 5B). Under these conditions, LNCaP and LNCaP-C4-2 cells showed a significant DAPI uptake rate (Fig. 5B). Interestingly, a very weak DAPI uptake rate was observed in the more aggressive PCa cell lines, which was similar to that observed under control conditions. This finding suggests that these cells do not present functional Cx hemichannels at their plasma membrane. In order to confirm whether or not the DAPI uptake was incorporated into the PCa cell lines via Cx hemichannels, 200 µM lanthanum (La^3+^) was added to the DCSF. Upon treatment with La^+ 3^, a significant reduction in DAPI uptake rate was observed in LNCaP and LNCaP-C4-2 PCa cell lines (Fig. 5C). However, the effect of La^3+^ on LNCaP-C4-2 was smaller when compared to LNCaP cells, which suggests that LNCaP-C4-2 cells in addition to hemichannels present other ways by which DAPI can be incorporated into the cells. Then, we subtracted the rate of dye uptake observed under control condition to that observed in DCFS, which gave us an estimate of the amount of DAPI incorporated by the PCa cells through hemichannels (Fig. 5D). Collectively, these analyzes revealed that there was a negative correlation between the hemichannel activity and the degree of aggressiveness of PCa cell lines.

### Cellular localization of Cx26, Cx32 and Cx43 in prostate Cancer cell lines

**Fig. 6:**
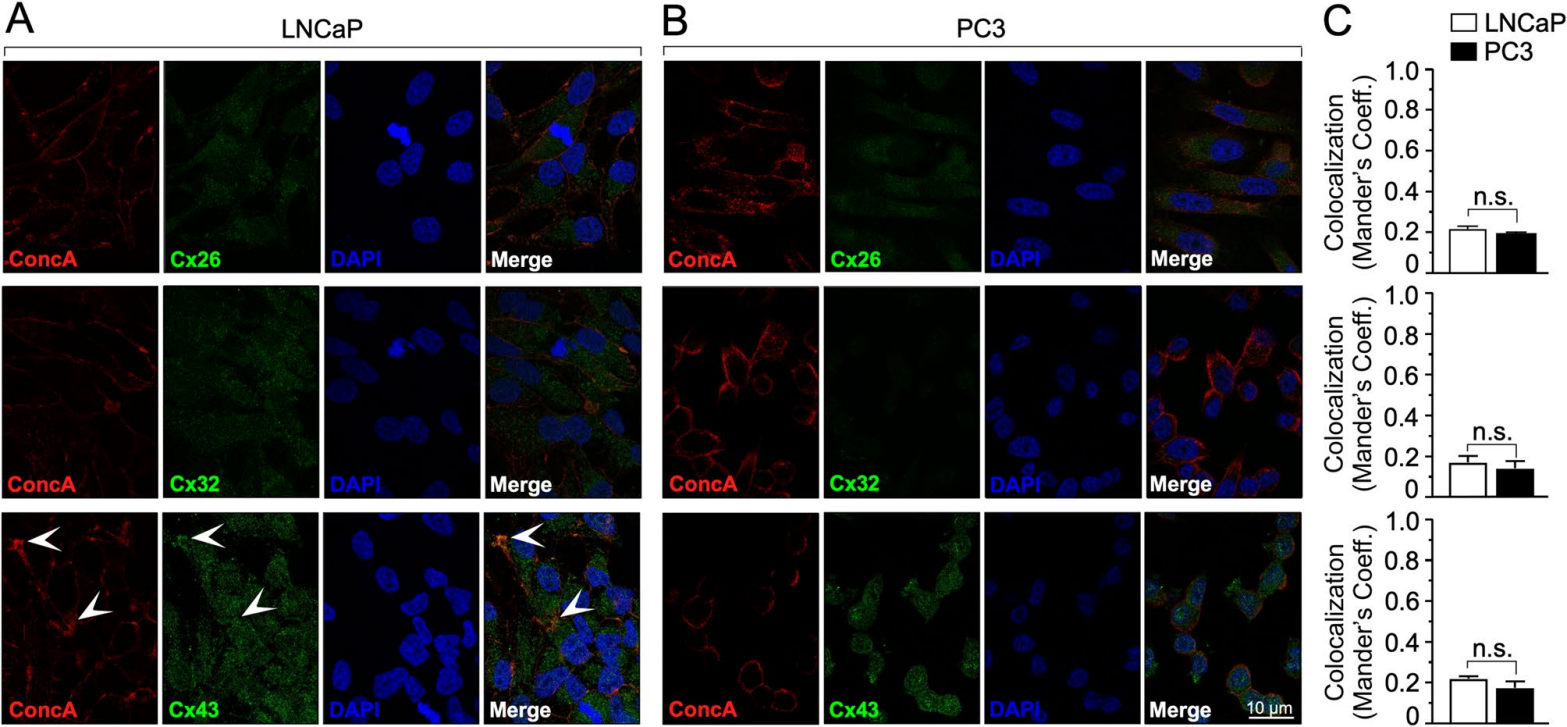
Cellular localization of connexins in PCa cells. Immunofluorescence analyses of Cx26, Cx32, and Cx43 in LNCaP (**A**) and PC3 (**B**) cell lines. Concanavalin A (ConcA) stained plasma membrane and DAPI stained nuclei of PCa cells. White arrow heads indicate focal areas of ConcA-Cx43 colocalization. White size bar: 10 μm. (**C**) Mander’s coefficient analysis was performed to compare ConcA-Cxs colocalization in LNCaP (white bars) and PC3 (black bars) cell lines. Mann Whitney test was utilized for statistical analyses. n.s. = not statistically significant

Considering that, at the protein level, only connexins Cx26, Cx32, and Cx43 exhibited a correlation with the aggressiveness of PCa cell lines. Our objective was to explore the cellular localization of these connexins in PCa cells. To achieve this, we focused on the LNCaP and PC-3 cell lines due to their moderate and low functionality in both, GJC and hemichannels, respectively (Figs. 3, 4 and 5). We conducted immunofluorescence analyses of the different connexins using confocal laser scanning microscopy (Leica SP8) (Fig. 6A-B). To visualize the cell membrane, cells were pre-stained with biotinylated concanavalin A (Fig. 6A-B, ConcA). Nuclei were stained using DAPI (Fig. 6A-B, DAPI). Consistent with our functional analyses, our findings revealed that the less aggressive LNCaP cells primarily expressed connexins Cx26 and Cx32 at cytoplasmic levels (Fig. 6A, Cx26 and Cx32). Cx43 expression in LNCaP cells was also predominantly cytoplasmic (Fig. 6A, Cx43), with occasional observations of Cx43 localization at the plasma membrane (Fig. 6A, Cx43, indicated by white arrowheads). Conversely, PC-3 cells displayed expression of Cx26 and Cx43 at cytoplasmic levels (Fig. 6B, Cx26 and Cx43), while consistent with our western blot analyses, minimal to no expression of Cx32 was detected in this cell line (Fig. 6B, Cx32). To assess the level of colocalization between the membrane marker, concanavalin A, and the various connexins in LNCaP and PC3 cell lines, we employed Manders’ analysis (Fig. 6C). Our results indicated no significant differences in concanavalin A-connexins (Cx26, Cx32, and Cx43) colocalization between LNCaP and PC3 cell lines (refer to Fig. 6C).

## Discussion

More than 50 years ago, loss of direct intercellular communication was first associated with cancer development and progression [9]. Since then, many studies have supported the concept that Cxs represent tumor suppressors [12]. In PCa, Cxs research has shown contradictory evidence regarding the biological role of some of the isoforms in the early and more advanced stages of the disease. Our study primarily analyzed the expression of Cx26, Cx32 and Cx43, because these isoforms have been described to be expressed in benign prostate epithelium and during prostate cancer progression [26, 27]. In addition, we also analyzed the expression of Cx37, Cx40 and Cx45 because these Cxs have been reported to be expressed in other cancer cell models [38–42], but have not been analyzed in PCa.

Our qRT-PCR results indicated that Cx26 mRNA is expressed at the highest level in the highly aggressive PC-3 cell line, when compared to the other PCa cell lines. This result might initially suggest that Cx26 could play a role in the advanced stages of PCa. However, when Cx26 protein levels were analyzed in the corresponding PCa cell lines, an opposite relationship was observed. In addition, opposite results were also observed when comparing Cx37 and Cx45 mRNA and protein expression levels in PCa cells (Figs. 1 and 2).

Similar to what was described by Tate et al. [28], we detected high levels of Cx26 protein in LNCaP cells. The fact that in our study LNCaP and LNCaP-C4-2 cell lines express high amounts of Cx26 protein, despite having low amounts of mRNA, could be explained by specific regulation of Cx26 expression at the post-transcriptional level. Protein synthesis is determined by the balance between mRNA degradation and stability, which is controlled by binding proteins or non-coding RNA to specific sequences within the mRNA [43]. In this case, it could be favoring Cx26 mRNA stabilization in these cell lines and, therefore, allowing a greater protein synthesis. Another explanation for this controversy might be related to the efficiency of Cx26 translation in PCa cell lines, which may result in high amounts of Cx26 protein despite having low amounts of Cx26 mRNA. Most mRNAs are translated by a cap-dependent canonical mechanism. However, some mRNAs might contain internal ribosome entry sites (IRES) that allow direct binding to the ribosome and their cap-independent translation. This mechanism has been reported in cancer cells under stress, apoptosis, among other conditions [13, 43]. Moreover, previous studies indicated that Cx26 mRNA contains IRES elements that induce its cap-independent translation in human pancreatic cancer cells [44].

At the protein level, we observed a clear inverse relationship between the expression of Cx32 and the aggressiveness of PCa cell lines. This finding aligns with prior research in human samples where Cx32 loss was associated with more advanced stages of prostate cancer [24]. Similarly, in hepatic cancer animal models treated with MitoQ [45], and in liver cancer, Cx32 has been shown to negatively regulate the expansion of liver cancer stem cells and, as expected, was positively correlated with patient survival [46]. Furthermore, in two breast cancer cell lines (MCF-7 and Hs578T), the enhancement of Cx32 expression resulted in reduced migration and invasion potentials [47]. Collectively, these examples underscore Cx32 as a broad-spectrum tumor suppressor, and PCa is no exception.

In the case of Cx43 mRNA and protein expression, our results indicated that the less aggressive PCa cell lines, LNCaP and LNCaP-C4-2, showed the highest expression of the mRNA and mRNA and protein, respectively, when compared to the more aggressive PCa cell lines, Du-145 and PC-3. These results contradict the results reported by Zhang et al. [15], which showed that the expression of Cx43, at the mRNA and protein levels, was positively associated with an increase in PCa malignancy. This controversy might be explained by differences in experimental conditions between both groups, such as using cells at different cell culture passages and/or confluences. In our case, our cell line models were used at very low passages (passage 1–5) after been commercially obtained from ATCC and all experiments were conducted with cells at no more than 80% confluence, though results were consistent even under higher confluence conditions (data not shown).

In addition, we focus on the analysis on the amount of mRNA and protein levels of Cx37, Cx40 and Cx45, which have not been studied in PCa, but have been described in other types of human neoplasms. A previous study showed that the C1019T polymorphism in the Cx37 gene correlates with the occurrence of gastric positive infection with *Helicobacter Pylori.* Subsequently, it was demonstrated that, by using a siRNA against Cx37 in subcutaneous gastric tumors grown in mice, tumor cell apoptosis was promoted [39, 48]. Other groups have found overexpression of Cx45 in clinical specimens of patients with acute myeloid leukemia (AML) [36], and oral squamous cell carcinoma (OSCC) [49]. We observed a positive relationship between the amount of Cx37 and Cx45 mRNAs and the aggressiveness of PCa cell lines (Figs. 1 and 2). However, Cx40 mRNA showed no clear relationship with the level of aggressiveness in PCa cell lines; LNCaP-C4-2 and PC-3 cell lines showed the highest level of expression of Cx40 mRNA. Once again, these differences observed at the mRNA level were not replicated at the protein level, observing a non-significant association between the expression level of the Cx37, Cx40 and Cx45 proteins and the degree of aggressiveness of PCa cell lines. These results show the need to unify criteria for the analysis of the expression of these proteins, considering aspects directly related to the cell types and their culture conditions.

Subsequently, we evaluated the ability of the different PCa cell lines to form functional GJCs. Interestingly, only the less aggressive PCa cell line, LNCaP, were able to moderately transfer the LY, which indicates that these cells possess some functional GJCs. Our results indicate that PCa cell lines that show greater tumor aggressiveness lose their intercellular communication mediated by GJs, which is in accordance with most of the functional studies carried out in cancer cells. In fact, Mehta et al. [27] analyzed cell-to-cell communication through GJCs in Du-145 and PC-3 cell lines, using the LY dye, and found similar results.

The moderate GJIC activity in the LNCaP cell line correlated with the high levels of expression of Cx26 and Cx43 observed in this cell line, which suggests that the intercellular channels could be constituted by one or both of these Cxs. Furthermore, the fact that only LY was transferred, can shed light on the type of Cx isoform involved in this communication. GJC have been proposed to exhibit Cx-type specific permeabilities to different substances. Ek-Vitorin et al. [50]. demonstrated that the negatively charged LY dye permeates through GJC composed of different Cxs types in the following the order of efficiency: Cx43 > Cx45 > Cx26 > Cx40, which suggests that Cx43 could be involved in GJIC in LNCaP cells. Our immunofluorescence analyses also suggest Cx43 as a potential mediator of the moderate GJIC activity observed in LNCaP cells. Even though we used two permeability tracers of different charges and sizes (LY: -2 and 443 kDa; Dapi: +1 and 279 kDa), adding more fluorescent tracers that differ in size or ionic charge, load, and stiffness to the functional study of GJs could help to solve this question with greater precision.

In the last decade, it has been found that hemichannels participate in various cellular processes, such as modulation of cell proliferation and survival [31]. However, their possible role in tumor growth has received less attention. Khalil et al. [30]. demonstrate that Cx43 hemichannels are functional and permit nucleotides and nucleosides release during the invasion process of breast cancer cells. In PCa cell models, the functionality of hemichannels formed by Cxs has not been evaluated. In our study, we measured the uptake of DAPI under basal conditions and in the presence of a medium without Ca^+ 2^ or Mg^+ 2^ as a stimulus to increase the open probability of Cx hemichannels [51, 52]. The fact that we did not observe a large amount of DAPI uptake under basal conditions is in accordance with the conservative concept that the hemichannels remain preferentially closed during cell-resting conditions [53]. In presence of medium lacking divalent cations, significant differences in dye uptake were observed in PCa cell lines. Du-145 and PC-3 cell lines showed low DAPI uptake, suggesting that the more aggressive PCa cell lines display low or no hemichannel activity. La^+ 3^ blocked DAPI uptake in the LNCaP PCa cell line by less than half. Nonetheless, in LNCaP-C4-2 cells, an effect of the La^+ 3^ was not visualized. Together, these results indicate that from all PCa cell lines analyzed, only LNCaP cells showed evident, but still moderate, hemichannel activity in vitro. This observation correlated with the findings at the level of GJIC and the expression patterns of Cx26 and Cx43 in LNCaP cells. More specifically, it matched the punctate localization of Cx43 at the plasma membrane of LNCaP cells, as determined by immunofluorescence. The fact that the highly aggressive, Du-145 and PC-3, PCa cell lines do not have functional GJC or hemichannel activity but do express certain Cxs such as Cx26, Cx43, Cx40 and Cx45 suggests that these isoforms could be fulfilling roles that could be independent of their capacity to form channels. Consequently, our immunofluorescence analyses revealed that Cx26 and Cx43 localized mostly at the cytoplasmic level in PC3 cells. Several studies have implicated a potential channel-independent activity of Cxs in supporting cell growth, differentiation, tumorigenesis, and apoptosis. Although, the mechanistic aspects of these actions remain poorly characterized [54, 55]. This channel-independent roles of Cxs have also been implicated in the regulation of migration and proliferation through direct Cx-protein interactions such as interactions of Cxs with the cytoskeleton. As an example, Cx43 induces migration of LNCaP cells that were exposed with osteoblasts-conditioned media, in a GJIC- and HC-independent manner [56].

Cxs are implicated in the activation or repression of the epithelial-mesenchymal transition (EMT) program in cancer cells [57, 58]. It is generally believed that connexins regulate EMT either as monomers or through GJs [59, 60]. For instance, Cx32 expression is downregulated in hepatocellular carcinoma (HCC) cells during carcinogenesis, and this decrease in Cx32 induces EMT by activating the Wnt/β-catenin/Snail pathway, leading to high invasion and metastasis characteristics in HCC cells [59]. Conversely, overexpression of Cx26 or Cx43 inhibits the proliferation of breast cancer cells in vitro and reduces tumorigenicity in vivo, promoting the transition from mesenchymal to epithelial states [61]. Additionally, overexpression of Cx43 increases the expression of E-cadherin and ZO-1 epithelial markers, while downregulation of Cx43 increases the expression of the N-cadherin mesenchymal marker in MDA-MB-231 cell lines [58, 62]. These findings indicate that connexins play a crucial role in regulating EMT in cancer progression and metastasis, highlighting their potential as biomarkers for cancer treatment and prognosis.

Even though most studies have focused on determining the channel-independent roles of Cx43 and 32 in cancer, our results also pointed out to Cx40 or Cx45 as potential candidates for modulating, in a GJC- and hemichannel-independent manner, the biology of highly aggressive PCa cell lines. Consequently, future studies should focus on evaluating the role of Cx40 and Cx45 in the progression of PCa and whether or not these proteins can affect cell proliferation and/or metastatic potential.

## Data Availability

The materials described in the manuscript, including all relevant raw data, will be freely available to any scientist wishing to use them for non-commercial purposes.
